# Dental Caries and Periodontal Disease in Brazilian Children and Adolescents with Cerebral Palsy

**DOI:** 10.3390/ijerph120100335

**Published:** 2014-12-29

**Authors:** Andreia M. R. Cardoso, Lays N. Gomes, Clara Regina D. Silva, Renata de S. C. Soares, Mauro Henrique N. G. de Abreu, Wilton W. N. Padilha, Alessandro L. Cavalcanti

**Affiliations:** 1 Department of Dentistry, State University of Paraiba, Campina Grande, PB, 58429-500, Brazil; E-Mails: andreiamedeiros29@yahoo.com.br (A.M.R.C.); drarenatacoelho@gmail.com (R.S.C.S.); 2 Department of Dentistry, Federal University of Paraiba, João Pessoa, PB, 58051-900, Brazil; E-Mails: laysnobrega@yahoo.com.br (L.N.G.); clarareginads@yahoo.com.br (C.R.D.S.); 3 Department of Community and Preventive Dentistry, Universidade Federal de Minas Gerais, Belo Horizonte, MG, 31270-901, Brazil; E-Mail: maurohenriqueabreu@ig.com.br; 4 Postdoctoral fellow, State University of Paraiba, Campina Grande, PB, 58429-500, Brazil; E-Mail: wiltonpadilha@yahoo.com.br

**Keywords:** dental caries, periodontal index, cerebral palsy, epidemiology

## Abstract

The aim of the present study was determine the prevalence and factors associated with dental caries and periodontal disease in Brazilian children and adolescents with cerebral palsy (CP). This is a cross-sectional study conducted with 80 patients ranging in age from 2 to 18 years old. Oral exams were conducted by an examiner with records of DMFT, dmft, Gingival Bleeding Index (GBI) and Community Periodontal Index (CPI). The statistical analysis used Poisson Regression with robust variance estimation (α = 0.05). The prevalence of dental caries was 59.3%, with DMFT and mean dmft of 1.71 ± 2.42 and 2.22 ± 3.23, respectively. The mean GBI was 22.44%, and in the CPI, the prevalence of gingival bleeding, calculus, shallow and deep pockets were 94.73%, 79.62%, 12.90% and 3.22%, respectively. The caregiver’s educational level of less than eight years were associated with the dental caries experience (PR = 1.439; 95%CI = 1.09–1.89). The periodontal alterations were associated with female sex (PR = 0.82; 95%CI = 0.69–0.97), caregiver’s educational level of less than eight years (PR = 1.15; 95%CI = 1.03–1.29), poor oral perception (PR = 0.89; 95%CI = 0.80–0.98), serious communication problem (PR = 0.87; 95%CI = 0.76–0.99) and athetoid type of CP (PR = 0.85; 95%CI = 0.75–0.97). The patients with CP presented high dental caries experience and periodontal alterations, which were associated with their demographic, socioeconomic, oral health perception and systemic information.

## 1. Introduction

Cerebral Palsy (CP) is defined as a group of movement and posture disorders, caused by a lesion that occurred in the developing fetal or infant brain [1]; its global prevalence is 2.1 per 1000 live births [2]. However, these motor dysfunctions frequently involve alterations in sensation, cognition, communication, behavior and epilepsy [1,3], causing partial or total reliance on the caregiver to perform daily activities, such as feeding, mobility and general and oral hygiene [4,5].

Individuals with CP presented high risk for developing oral disorders, particularly dental caries and periodontal alterations [6]. The more serious the neurological deficiency in people with CP, the greater the risk of dental caries [4,7,8], with loss of executive function and functional deficit directly proportional to the chance to develop dental caries [9].

Dental caries, besides presenting high prevalence in children and adolescents with CP [4,8,10,11,12], also represented a negative impact on their quality of life in terms of oral health [5,13]. The individuals with CP also showed high prevalence of periodontal alteration [10,14,15,16,17], related to factors arising from the injury in the nervous system, such as mental deficiency [16,17] and orofacial motor dysfunction [18], which may hinder daily oral hygiene and hence increase dental biofilm formation. However, these results cannot be generalized and tend to vary with the country/region [19].

The literature has studies conducted with CP individuals which analyze the association of dental caries and periodontal alteration with demographic [12,15,20], socioeconomic [15], systemic [11,12,18] and behavioral [4,12,15,20] factors, but no evaluation of aspects related to perception, dental service access and communication has been found. Thus, the objective of this study was to determine the prevalence and factors associated with dental caries and periodontal alterations in children and adolescents with CP.

## 2. Materials and Method

### 2.1. Studied Population

This cross-sectional study was conducted in the city of Campina Grande, Paraíba. This city has 385,213 inhabitants [21] and it is located in the mesoregion of the *agreste* region, state of Paraíba, northern Brazil. The study was conducted in a reference center for children and adolescents with CP, named *Associação de Pais e Amigos de Excepcionais* (APAE). This institution offers therapeutic and educational activities and social integration to these individuals. The studied population included 97 children and adolescents with diagnosed CP, between two and 18 years old, and their respective caregivers (*n* = 97). The refusal to participate in the study and non-attendance for clinical examination after three successive attempts was considered a loss.

### 2.2. Eligibility Criteria

The study included children and adolescents between two and 18 years old with CP and their caregivers. All individuals included in present study had a clinical medical diagnosis of cerebral palsy—G80 (International Classification of Diseases and Related Health Problems 10th, ICD-10) [22]. Caregivers were considered as individuals of 18 years old or above, who were responsible for making decisions and daily activities of these people with CP [11]. Children and adolescents whose caregivers had been in this occupation for less than three months were excluded.

### 2.3. Calibration and Training Process

The calibration process involved two stages: a theoretical stage and a clinical stage. A dental surgeon and a gold standard specialized in Pediatric Dentistry participated in the process. During the theoretical activities, the diagnostic criteria for dental caries and periodontal alterations were discussed [23]. In the clinical stage, 40 children and adolescents without neurological deficiency were examined at a preschool and a public school. Kappa values to evaluate the examiner’s agreement in the dental caries and periodontal alteration diagnosis were 0.75 and 0.66, respectively.

### 2.4. Data Collection

Data collection was conducted by one examiner and one data recorder. At first, clinical data with information related to the CP location [1] and type of neuromuscular dysfunction (Spastic, Athetoid, Ataxic and Mixed) [1] were collected from patients charts. After that, in a service room at APAE, a clinical form was filled in a face-to-face conversation with caregivers. The form presented the following information: socioeconomic aspects (children’s gender and age, caregiver’s educational level, the family’s monthly income classified according to the minimum wage in Brazil), perception of general and oral health of the child/adolescent, dental service access (medical visit, service sector and any difficulty with medical visit), behavioral aspects (diet consistency, number of snacks, tooth brushing frequency and any difficulty with tooth brushing) and systemic information (use of medication and presence of epilepsy).

Clinical exams were conducted, with the patient sitting on own wheelchair or a traditional chair, after tooth brushing, under supervision [4,13]. To conduct the exam, the examiner used: a 250-lumen LED (Light Emitting Diode) light bulb coupled to the head, flat dental mirrors, periodontal probes (Community Periodontal Index—CPI, Trinity Ind. Com. Ltda, São Paulo, SP, Brazil), mouth openers, wooden spatulas and disposable gauzes [23].

In the general clinical examination, the following was observed: type of breathing [7], communication capability [24] and presence of labial hypotonia [25]. Lip hypotonia was considered as the absence of lip sealing at rest and the vigorous contraction of the orbicular muscles of mouth to achieve sealing [25].

In the intraoral clinical examination, the following data were collected: DMFT and dmft indexes [23], GBI [26] for the age group of 2–6 years old, adapted with index teeth [14] and CPI [23] for the age group of 7–18 years old, for six index teeth [23,27].

### 2.5. Statistical Analysis

The analysis used descriptive statistics to characterize the sample. Bivariate and multivariate Poisson regression analysis with robust variance (*p* < 0.05) was used to determine the association between independent variables (socioeconomic, perception, dental service access, behavioral, clinical and oral) and dependent variables (dental caries and periodontal alterations), after categorization (Table 1).

**Table 1:** Categorization of variables.

Variable	Type of Record	In This Study
Gender	Gender of child or adolescent:	No adaptation
	1—Male; 2—Female	
Age group	Answer to question: “How old is the child or adolescent with CP?” (in years)	Categorized in the dental caries evaluation according to the type of dentition, as follows:
		1—2 to 5 years old
		2—6 to 11 years old
		3—12 to 18 years old.
		In the study on periodontal alterations, they were categorized according to the age group of indexes:
		1—2 to 6 years old
		2—7 to 18 years old.
Caregiver’s educational level	Answer to the question: “What’s your educational level (in years)?”	Categorized as:
		1—8 years or less
		2—more than 8 years.
Monthly family income	Ratio of combined family income, in US $, received last month by everyone who lives in the house and number of people who live in the house?	Categorized by median as:
		1—up to US$101.80
		2—above US$101.80
Perception of general health	Caregiver’s perception of general health of child/adolescent: 1—Excellent; 2—Good; 3—Bad; 4—Very bad.	Categorized as:
		1—Good (excellent and good)
		2—Bad (bad and very bad).
Perception of oral health	Caregiver’s perception of oral health of child/adolescent: 1—Excellent; 2—Good; 3—Bad; 4—Very bad.	Categorized as:
		1—Good (excellent and good)
		2—Bad (bad and very bad).
Visit to dentist	Answer to question: “Has the child or adolescent ever been to the dentist office?”	No adaptation
	0—No; 1—Yes; 9—Doesn’t know.	
Dental Service Sector	Answer to question: “Where was the last CP child dental visit?” 1—Public service; 2—Private service; 3—Dental plan; 4—Other; 8—Not applicable; 9—Doesn’t know	Categorized as:
		1—Public service
		2—Private service (private service and dental plan).
Difficult access to service	Answer to question: “Is it difficult for you to have access to dental service for the child/adolescent?”	No adaptation
	0—Não; 1—Sim; 9—Doesn’t know.	
Food consistency	Food consistency of child/adolescent: 1—Solid, 2—Semisolid and 3—Liquid.	Categorized as:
		1—Solid (solid and semisolid)
		2—Liquid.
Number of snacks	Answer to question: “How many snacks does the child/adolescent have (excluding breakfast, lunch and dinner)?”	Categorized as:
		1—3 times a day or less
		2—More than 3 times a day.
Brushing frequency	Answer to question: “How often (daily) does brushing of child/adolescent occur?”	Categorized as:
		1—Twice a day or less
		2—More than twice a day.
Difficulty to brush	Answer to question: “Do you find any difficulty while conducting oral hygiene of the child/adolescent?”	No adaptation
	0—No; 1—Yes; 9—Doesn’t know.	
Dental caries experience	In records of DMFT or dmft indexes, the total is 1 or above when adding DMFT or dmft indexes.	Categorized as:
		0—Absent dental caries experience
		1—Present dental caries experience.
Periodontal alterations	In records of GBI, percentage of 1% or above. In records of CPI, codes 1 and 4.	Categorized as:
		0—Absence of periodontal alterations;
		1—Presence of periodontal alterations.
Labial hypotonia	The absence of labial seal in the clinical evaluation of lips:	No adaptation
	0—Absent; 1—Present; 9—No information.	
CP location	CP type, according to the affected parts of child/adolescent’s body:	No adaptation
	1—Tetraparesis, 2—Diparesis and 3—Hemiparesis.	
Type of CP dysfunction	Type of neuromuscular dysfunction of CP in the child/adolescent:	No adaptation
	1—Spastic, 2—Athetoid, 3—Ataxic and 4—Mixed.	
Epilepsy	Records of convulsive crises/epilepsy in clinical evaluation:	No adaptation
	0—Absent; 1—Present; 9—No information.	
Type of breathing	Type of breathing of child/adolescent:	No adaptation
	1—Nasal; 2—Mouth.	
Use of medication	Chronic medication use by the child/adolescent: 0—No; 1—Yes; 9—No information.	No adaptation for dental caries experience.
		For the study on periodontal alterations, the use of anticonvulsant was considered, categorized as:
		0—No
		1—Yes.
Communication capability	Communication capability of child/adolescent:	No adaptation
	0—Normal, 1—Mild deficiency, 2—Moderate deficiency, 3—Serious deficiency.	

A hierarchical approach procedure [28] was used in the multivariate regression model to select variables that reached *p* < 0.20 in the bivariate analysis. The analysis was conducted at six levels, from distal to proximal determinants: (1) socioeconomic, (2) health perception, (3) dental service access, (4) behavioral, (5) oral, and (6) systemic. The variables of *p* < 0.05 in the adjusted analysis were kept in the final regression model. All tests were conducted in *Statistical Package for the Social Sciences* (SPSS for Windows, version 18.0, SPSS Inc, Chicago, IL, USA).

### 2.6. Ethical Aspects

This study followed ethical guidelines recommended by the Brazilian legislation and was approved by the Human Research Ethics Committee of the State University of Paraiba. All participants/guardians signed the informed consent form.

## 3. Results

The response rate was 82.5%, with 80 children and adolescents examined (42 male and 38 female participants). Seventeen recruited patients were not evaluated—three of them refused to participate and 14 did not attend the clinical examination. Regarding the sample, the most prevalent age group was 7–11 years old (46.3%), patients with tetraparesis (56.2%) and spasticity neuromuscular dysfunction (75.0%) (Table 2).

**Table 2:** Sample characteristics according to sex, age, CP location, type of disability, caregiver education and monthly family income.

Variables	Frequency	
	N	(%)
Sex		
Male	42	52.5
Female	38	47.5
Age of children		
2 to 6 years	22	27.5
7 to 11 years	37	46.3
12 to 18 years	21	26.2
CP location		
Tetraparesis	45	56.2
Diparetic	23	28.8
Hemiparesis	12	15.0
Type of disability		
Spastic	60	75.0
Athetoid	18	22.5
Unrated	2	2.5
TOTAL	80	100.0

Dental caries experience was 71.3% and the presence of untreated dental caries was 59.3%. The mean values of DMFT and dmft were 1.71 ± 2.42 and 2.22 ± 3.23, respectively (Table 3). In the periodontal evaluation, 89.9% presented alterations. Mean GBI was 22.44%, and in the CPI examination, the prevalences of gingival bleeding, calculus, shallow and deep pockets were 94.73%, 79.62%, 12.90% and 3.22%, respectively (Table 4). Of the five patients with shallow or deep periodontal pockets, three were making use of anticonvulsant medication.

**Table 3:** DMFT and dmft indexes and components, measured in children and adolescents with CP.

Indexes and Components	Mean and Standard Deviation	Median	Min.–Max.
DMFT + dmft (*n* = 80)	2.65 ± 3.15	1.5	0–14
DMFT (*n* = 65)	1.70 ± 2.42	1.0	1–11
D	1.32 ± 2.37	1.0	0–11
M	0.20 ± 0.77	0.0	0–5
F	0.43 ± 1.34	0.0	0–7
dmft (*n* = 46)	2.22 ± 3.23	1.0	0–14
D	1.54 ± 2.59	3.5	0–14
M	0.19 ± 1.06	0.0	0–7
F	0.19 ± 0.65	0.5	0–4

**Table 4:** Distribution of children and adolescents with CP according to the presence of periodontal changes in CPI and GBI measured.

Variables	Frequency	
	N	%
Presence of periodontal changes (*n* = 79) *		
Yes	71	89.9
No	8	10.1
Maximum CPI (*n* = 57)		
Healthy	3	5.3
Bleeding	11	19.3
Calculus	38	66.7
Shallow pocket	4	7.0
Deep pocket	1	1.8
GBI (%; *n* = 22)		
0–10	8	36.4
11–25	9	40.9
Above 25	5	22.7

* A child did not allow the clinical examination of periodontal changes.

In the bivariate analysis, independent variables: caregiver’s educational level, perception of general and oral health and communication capability were associated with dental caries experience (*p* < 0.05) (Table 5). These variables, plus gender, family income and CP location, were incorporated into the multivariate model (*p* < 0.20). The caregiver’s educational level (PR = 1.43; 95% CI = 1.09–1.89) remained in the final Poisson regression model for dental caries experience (Table 5).

**Table 5:** Distribution of children and adolescents with CP in Poisson regression bivariate and multivariate models for dental caries experience (DMFT + dmft > 1) and independent variables.

Variable	Dental Caries Experience		Bivariate		Multivariate	
	Absent n (%)	Present n (%)	*p*-Valor	Unadjusted RP * (CI 95%)	*p*-Value	Adjusted RP ^†^ (CI 95%)
**LEVEL 1—SOCIOECONOMIC CHARACTERISTICS**						
**Sex**						
Male	16 (38.1)	26 (61.9)		0.75 (0.57–1.00)	-	-
Female	7 (18.4)	31 (81.6)	0.054	1.00	-	-
**Age**						
2 to 5 years	10 (45.5)	12 (54.5)	0.258	0.76 (0.47–1.21)	-	-
6 to 11 years	7 (18.9)	30 (81.1)	0.426	1.13 (0.83–1.55)	-	-
12 to 18 years	6 (28.6)	15 (71.4)		1.00	-	-
**Caregiver education**						
≤4 schooling	4 (14.8)	23 (85.2)	0.030	1.32 (1.02–1.71)	0.010	1.43 (1.09–1.89)
>4 schooling	19 (35.8)	34 (64.2)		1.00		1.00
**Monthly family income**						
≤US$101.80	9 (22.0)	32 (78.0)	0.177	1.21 (0.91–1.62)	-	-
>US$101.80	14 (35.9)	25 (64.1)		1.00	-	-
**LEVEL 2—HEALTH PERCEPTION CHARACTERISTICS**						
**General health perception**						
Good	23 (29.9)	54 (70.1)		0.70 (0.60–0.81)	-	-
Poor	0 (0.0)	3 (100.0)	0.001	1.00	-	-
**Oral health perception**						
Good	20 (33.9)	39 (66.1)		0.77 (0.59–0.99)	-	-
Poor	3 (14.3)	18 (85.7)	0.44	1.00	-	-
**LEVEL 3—ACCESS TO DENTAL SERVICES**						
**Visit to dentist**						
Yes	18 (27.7)	47 (72.3)	0.682	1.08 (0.73–1.59)	-	-
No	5 (33.3)	10 (66.7)		1.00	-	-
**Type of service**						
Public	12 (24.0)	38 (76.0)	0.294	1.26 (0.81–1.97)	-	-
Private	6 (40.0)	9 (60.0)		1.00	-	-
**Difficulty for appointment**						
Yes	11 (25.0)	33 (75.0)	0.667	1.06 (0.80–1.40)	-	-
No	10 (29.4)	24 (70.6)		1.00	-	-
**LEVEL 4—BEHAVIORAL CHARACTERISTICS**						
**Diet consistency**						
Solid	17 (26.2)	48 (73.8)	0.353	1.23 (0.79–1.90)	-	-
Liquid	6 (40.0)	9 (60.0)		1.00	-	-
**Number of meals**						
≤3 meals	22 (29.3)	53 (70.7)		0.94 (0.52–1.69)	-	-
>3 meals	1 (25.0)	3 (75.0)	0.842	1.00	-	-
**Tooth brushing frequency**						
≤2 times	11 (26.2)	31 (73.8)	0.547	1.07 (0.81–1.42)	-	-
>2 times	12 (31.6)	26 (68.4)		1.00	-	-
**Difficulty during brushing**						
Yes	14 (28.6)	35 (71.4)	0.965	1.00 (0.75–1.34)	-	-
No	9 (29.0)	22 (71.0)		1.00	-	-
**LEVEL 5—ORAL CHARACTERISTICS**						
**Periodontal changes**						
Absent	4 (50.0)	4 (50.0)		0.74 (0.38–1.43)	-	-
Present	18 (25.4)	53 (74.6)	0.374	1.00	-	-
**Labial hypotonia**						
Absent	7 (38.9)	11 (61.1)		0.82 (0.55–1.22)	-	-
Present	16 (25.8)	46 (74.2)	0.338	1.00	-	-
**LEVEL 6—SYSTEMIC CHARACTERISTICS**						
**CP location**						
Tetraparesis	15 (33.3)	30 (66.7)		0.80 (0.57–1.10)	-	-
Diparetic	6 (26.1)	17 (73.9)	0.530	0.88 (0.62–1.26)	-	-
Hemiparesis	2 (16.7)	10 (83.3)	0.181	1.00	-	-
**Type of disability**						
Spastic	16 (26.7)	44 (73.3)	0.370	1.20 (0.80–1.78)	-	-
Athetoid	7 (38.9)	11 (61.1)		1.00	-	-
**Epilepsies**						
Absent	8 (25.8)	23 (74.2)	0.638	1.06 (0.80–1.41)	-	-
Present	15 (30.6)	34 (69.4)		1.00	-	-
**Type of breathing**						
Nasal	8 (38.1)	13 (61.9)		0.83 (0.57–1.19)	-	-
Mouth	15 (25.4)	44 (74.6)	0.320	1.00	-	-
**Use of medication**						
Yes	5 (22.7)	17 (77.3)	0.434	1.12 (0.84–1.49)	-	-
No	18 (31.0)	40 (69.0)		1.00	-	-
**Communication capability**						
Normal	0 (0.0)	6 (100.0)	0.049	1.80 (1.01–3.22)	-	-
Mild deficiency	3 (15.0)	85 (17.0)		1.00	-	-
Moderate deficiency	16 (35.6)	29 (64.4)	0.174	1.53 (0.82–2.82)	-	-
Serious deficiency	4 (44.4)	5 (55.6)	0.641	1.16 (0.62–2.16)	-	-

* Poisson regression not adjusted for independent variables and caries experience. **^†^** Multivariate Poisson regression adjusted for caries experience and socioeconomic, demographic, perception characteristics, access to dental services, behavioral, oral and systemic characteristics (independent variables) by the hierarchical procedure.

Periodontal alterations, in the bivariate analysis, presented association with the following variables: gender, caregiver’s educational level, number of meals, caregiver’s perception of general and oral health, type of CP dysfunction and communication capability (*p* < 0.05; Table 6).

**Table 6:** Distribution of children and adolescents with CP in Poisson regression bivariate and multivariate models for the presence of periodontal changes and independent variables.

Variable	Periodontal Changes		Bivariate		Multivariate	
	Absent n (%)	Present n (%)	*p*-Valor	Unadjusted RP * (CI 95%)	*p*-Value	Adjusted RP ^†^ (CI 95%)
**LEVEL 1—SOCIOECONOMIC CHARACTERISTICS**						
**Sex**						
Male	7 (17.1)	34 (82.9)		0.85 (0.73–0.98)		0.82 (0.69–0.97)
Female	1 (2.6)	37 (97.4)	0.034	1.00	0.023	1.00
**Age**						
2 to 6 years	5 (22.7)	17 (77.3)		0.81 (0.64–1.03)	-	-
7 to 18 years	3 (5.3)	54 (94.7)	0.089	1.00	-	-
**Caregiver education**						
≤8 schooling	0 (0.0)	27 (100.0)	0.005	1.18 (1.05–1.32)	0.013	1.15 (1.03–1.29)
>8 schooling	8 (15.4)	44 (84.6)		1.00		1.00
**Monthly family income**						
≤US$101.80	4 (9.8)	37 (90.2)	0.910	1.00 (0.87–1.17)	-	-
>US$101.80	4 (10.5)	34 (89.5)		1.00	-	-
**LEVEL 2—HEALTH PERCEPTION CHARACTERISTICS**						
**General health perception**						
Good	8 (10.5)	68 (89.5)		0.89 (0.82–0.96)	-	-
Poor	0 (0.0)	3 (100.0)	0.005	1.00	-	-
**Oral health perception**						
Good	8 (13.8)	50 (86.2)		0.89 (0.82–0.96)		0.89 (0.80–0.99)
Poor	0 (0.0)	21 (100)	0.005	1.00	0.028	1.00
**LEVEL 3—ACCESS TO DENTAL SERVICES**						
**Visit to dentist**						
Yes	6 (9.2)	59 (90.8)	0.621	1.05 (0.84–1.32)	-	-
No	2 (14.3)	12 (85.7)		1.00	-	-
**Type of service**						
Public	3 (6.0)	47 (94.0)	0.229	1.17 (0.90–1.52)	-	-
Private	3 (20.0)	12 (80.0)		1.00	-	-
**Difficulty for appointment**						
Yes	4 (9.3)	39 (90.7)	0.729	1.02 (0.88–1.20)	-	-
No	4 (11.8)	30 (88.2)		1.00	-	-
**LEVEL 4—BEHAVIORAL CHARACTERISTICS**						
**Diet consistency**						
Solid	7 (10.9)	57 (89.1)		0.95 (0.81–1.12)	-	-
Liquid	1 (6.7)	14 (93.3)	0.567	1.00	-	-
**Number of meals**						
≤3 meals	8 (10.8)	66 (89.2)		0.89 (0.82–0.96)	-	-
>3 meals	0 (0.0)	4 (100.0)	0.005	1.00	-	-
**Tooth brushing frequency**						
≤2 times	3 (7.3)	38 (92.7)	0.397	1.06 (0.91–1.24)	-	-
>2 times	5 (13.2)	33 (86.8)		1.00	-	-
**Difficulty during brushing**						
Yes	4 (12.9)	27 (87.1)		1.00	-	-
No	4 (8.3)	44 (91.7)	0.531	1.05 (0.89–1.23)	-	-
**LEVEL 5—ORAL CHARACTERISTICS**						
**Periodontal changes**						
Absent	4 (18.2)	18 (81.8)		0.88 (0.71–1.08)	-	-
Present	4 (7.0)	53 (93.0)	0.231	1.00	-	-
**Labial hypotonia**						
Absent	3 (16.7)	15 (83.3)		0.90 (0.72–1.13)	-	-
Present	5 (8.2)	56 (91.8)	0.388	1.00	-	-
**LEVEL 6—SYSTEMIC CHARACTERISTICS**						
**CP location**						
Tetraparesis	2 (4.4)	43 (95.6)	0.304	1.14 (0.88–1.48)	-	-
Diparetic	4 (18.2)	18 (81.8)		0.98 (0.71–1.35)	-	-
Hemiparesis	2 (16.7)	10 (83.3)	0.901	1.00	-	-
**Type of disability**						
Spastic	8 (13.3)	52 (86.7)		0.86 (0.78–0.95)	-	0.85 (0.75–0.97)
Athetoid	0 (0.0)	17 (100.0)	0.005	1.00	0.017	1.00
**Epilepsies**						
Absent	5 (16.1)	26 (83.9)		0.86 (0.78–0.95)	-	-
Present	3 (6.3)	45 (93.7)	0.201	1.00	-	-
**Type of breathing**						
Nasal	3 (14.3)	18 (85.7)		0.93 (0.77–1.13)	-	-
Mouth	5 (8.6)	53 (91.4)	0.513	1.00	-	-
**Use of anticonvulsnts medications**						
Yes	5 (14.3)	30 (85.7)		0.92 (0.78–1.07)	-	-
No	3 (6.8)	41 (93.2)	0.297	1.00	-	-
**Communication capability**						
Normal	1 (16.7)	5 (83.3)		0.86 (0.76–0.97)		0.86 (0.61–1.19)
Mild deficiency	1 (5.0)	19 (95.0)	0.318	0.95 (0.85–1.05)	0.353	0.94 (0.80–1.09)
Moderate deficiency	6 (13.6)	38 (86.4)	0.317	0.83 (0.58–1.19)	0.439	0.87 (0.76–0.99)
Serious deficiency	0 (0.0)	9 (100.0)	0.014	1.00	0.035	1.00

* Poisson regression not adjusted for independent variables and caries experience. **^†^** Multivariate Poisson regression adjusted for caries experience and socioeconomic, demographic, perception characteristics, access to dental services, behavioral, oral and systemic characteristics (independent variables) by the hierarchical procedure.

In the multivariate model (Table 6), these variables were incorporated, plus child’s age (*p* < 0.20), remaining in the final model the association of periodontal alterations with female gender (PR = 0.82; 95% CI = 0.69–0.97), caregiver’s educational level less than eight years (PR = 1.15; 95% CI = 1.03–1.29), poor oral perception (PR = 0.89; 95% CI = 0.80–0.98), serious communication problem (PR = 0.87; 95% CI = 0.76–0.99) and athetoid type of CP (PR = 0.85; 95% CI = 0.75–0.97).

## 4. Discussion

Children and adolescents with CP present dental caries and periodontal alterations more frequently and more intensively, when compared to patients without neurological deficiency [4,10]. This study attempted to evaluate the prevalence and factors associated with dental caries and periodontal alterations in Brazilian children and adolescents with CP, and it is one of the few studies conducted in Brazil with the purpose to investigate, in these individuals, their association with socioeconomic, health perception, dental service access, systemic and oral factors.

In this study, dental caries experience was high, with similar results to those described in other studies in Brazil [11,15] and China [10]. The mean values of DMFT and dmft indexes were also high and similar to those described in India [16], Turkey [29], South Africa [30] and Brazil [15,18]. However, these results did not agree with the findings obtained from individuals in Saudi Arabia [20], China [10] and southern Brazil [4,11,12].

These divergences showed that prevalences of dental caries and DMFT and dmft indexes may vary with the country and region of a country, with such variability related to the age group, size, sample selection methods [10,12,15,20], although all of them have used the same criteria and indexes for dental caries diagnosis [23]. The high number of untreated dental caries found in the study agrees with some studies [10,11,12,15,20] and can be expected for children and adolescents with CP, considering the reports of low availability and dental service to patients with disabilities [31] and organization and geographical access problems of their caregivers [32].

The dental caries prevalence was also higher than the results described in other studies with pediatric population without neurological deficiency [33,34]. This high frequency in CP individuals is attributed to the presence of involuntary movements, pathological oral reflection, spasticity of muscles of mastication and food waste that may contribute to poor oral hygiene and, consequently, higher occurrence of dental caries [4,18].

In the pediatric population, the factors usually associated with dental caries described in the literature are gender [34], age [34], family income [35,36], educational level [37], toothache and as the main reason to see the dentist [38], respiratory problems [39], diet [4,38], oral hygiene [35,36], fluorosis [40], bruxism [39] and frequent gingival bleeding [36,38]. In this study, the dental caries experience was associated with the caregiver’s low educational level (less than eight years), a similar result to those obtained in a study conducted in Turkey [41], but that did not agree with other Brazilian studies [15,18]. However, comparisons between these studies should be made with precaution, due to their sample size and selection criteria.

The association of dental caries with the caregiver’s educational level confirms the assumption that the caregiver’s low educational level increases the probability of dental caries [37,42]. Caregivers with high educational level have better health knowledge and positive attitudes towards oral health, including better oral health habits and regular visits to dental surgeon [37,41]. For this reason, children present better oral health conditions [37].

Some studies that evaluated factors associated with dental caries in children and adolescents with CP found an association with increasing age [4,11,15], increasing caregiver wages [11], liquid consistency of diet [4], deficient oral hygiene [12,20], high values of salivary osmolality [8] and deficient orofacial motor skills [4,8].

Thus, the occurrence of dental caries in individuals with CP may be related to specific conditions of reduced motor coordination (which affects the practice of oral hygiene and diet consistency) and intellectual function, which makes effective personal or professional hygiene and oral care more difficult [6,8,9,12], as well as other determinants for the population in general, such as social inequality [6].

The prevalence of periodontal alterations was high. Younger children (2–6 years) presented moderate gingivitis [27]. In the CPI example, children and adolescents of 7–18 years presented bleeding and calculus as more frequent alterations. This high prevalence of periodontal alterations, especially bleeding and calculus, was also observed in India [16,17] and Brazil [15]. No study was found that used GBI in children and adolescents with CP. However, evaluations were conducted with the gingival index [43] that identified the presence of moderate inflammation in the group of patients with tetraparesis and spasticity [14], which were similar to the gingival inflammation classification and characteristics of the sample analyzed in this study.

Previous studies showed that the values of gingival bleeding and gingival hyperplasia were higher in children with CP [10,14]. This high frequency may be due to the same factors related to dental caries, which lead to biofilm buildup [10,14,15,16,17]. One of the explanations for this situation could be the difficulties to conduct daily oral hygiene, affected by alterations to intraoral sensitivity and orofacial motor dysfunction [4,18] and the absence of information about dental care [15]. Another related factor was the use of anticonvulsants (DPH, sodium valproate, and phenobarbital), as these medications induce gingival hyperplasia and constitute a predictive factor for periodontal diseases [10,14,15]. However, the use of anticonvulsants was not associated with the presence of periodontal alterations, as described in Table 5.

The Brazilian and international literature does not address the factors associated with periodontal alterations in children and adolescents with CP. In this study, the presence of periodontal alterations was associated with female gender, caregiver’s low educational level (less than eight years), caregiver’s poor perception of dental care of children and adolescents, serious communication problem and athetoid type of CP. The caregiver’s low educational level was also associated with this problem in children and adults with neurological problems, including CP [16,17], suggesting its influence on the caregiver’s attitudes, as reported for dental caries.

In contrast, studies that evaluated the factors associated with periodontal alterations exclusively in children with CP [14,15] showed association with increased age [15] and tetraparesis, when compared to the condition of hemiparesis [14]. These factors did not present any association in this study. However, comparisons should be made with precaution, due to different diagnostic procedures.

In this study, the association of periodontal alterations with female gender may be related to biological factors, such as hormone production [44], as the lifestyle of patients with CP is very similar, regardless of their gender, due to the fact that they present the same pathological behaviors and reflections [12,45].

The caregiver’s poor perception of the child or adolescent’s oral health was also associated with this problem. The presence of bleeding has been described as a factor directly associated with negative self-perception of oral health [46]. In general, the population classifies its oral health as poor in situations of clinical discomfort, deficient mastication or esthetic function, generating dissatisfaction to smile or speak [46]. Thus, although periodontal alterations are asymptomatic, they can cause gingival bleeding while brushing the CP patient’s teeth, suggesting to the caregiver that something is wrong.

Children and adolescents with CP that did not present any type of communication with their caregivers, classified as serious communication problem, were associated with high frequency of periodontal alterations. It suggests that a child’s difficulty to express feelings and understand the importance of satisfactory dental care provided by the caregiver increases the prevalence of this problem. Studies that evaluate this relation in these individuals have not been found in the literature.

The athetoid type of CP was also associated with periodontal alterations, indicating that continuous and uncontrolled movements of neck muscles result in excessive head movement [47] which consequently makes oral hygiene more difficult and increases the frequency of periodontal alterations.

This study has limitations that should be taken into account. For instance, the cross-sectional design does not allow to establish causal relations between the independent variables and the decay experience or periodontal alterations. In addition, just like previous studies, the participants of this study were from a rehabilitation institution; then, the results do not necessarily represent all individuals with CP. However, epidemiological studies with CP individuals with external validity are not found in the literature. In relation to periodontal examination, three adolescents who had periodontal pocket were making use of anticonvulsant medication, which can induce gingival overgrowth. Presence of bone loss could have been verified by radiographic examination. However, the absence of this equipment at APAE impaired the performance of this exam.

The dental caries experience and presence of periodontal alterations were associated with social (sex, caregiver’s educational level and perception) and/or systemic characteristics (type of CP dysfunction and communication capability) of children and adolescents with CP. Thus, the results of this study can guide dental surgeons while planning and conducting their strategies and clinical protocols for the provision of service to children and adolescents with CP, by addressing the problems of their social, systemic and oral hygiene scenarios, as well as their caregivers’ situations, and support administrators’ actions to expand oral health care to these individuals, for better oral health conditions and the quality of life of CP individuals.

The results of this study also indicate that interventions are required to increase the acceptance of children and adolescents with CP when dental care is provided by family and professional caregivers [6], and increase the effectiveness of oral problem prevention, to minimize curative or mutilating treatments.

## 5. Conclusions

Children and adolescents with CP presented high dental caries experience and periodontal alterations, and these problems were associated with presented demographic, socioeconomic, health perception and systemic characteristics.
